# Feasibility of relaxation along a fictitious field in the 2nd rotating frame (T_RAFF2_) mapping in the human myocardium at 3 T

**DOI:** 10.3389/fcvm.2024.1373240

**Published:** 2024-12-04

**Authors:** Joao Tourais, Maša Božić-Iven, Yidong Zhao, Qian Tao, Iain Pierce, Christian Nitsche, George D. Thornton, Lothar R. Schad, Thomas A. Treibel, Sebastian Weingärtner, Mehmet Akçakaya

**Affiliations:** ^1^ Imaging Physics, Delft University of Technology (TU Delft), Delft, Netherlands; ^2^ Computer Assisted Clinical Medicine, Medical Faculty Mannheim, Heidelberg University, Mannheim, Germany; ^3^ Mannheim Institute for Intelligent Systems in Medicine, Medical Faculty Mannheim, Heidelberg University, Mannheim, Germany; ^4^ Barts Heart Centre, Barts Health NHS Trust, London, United Kingdom; ^5^ Institute of Cardiovascular Science, University College, London, United Kingdom; ^6^ Electrical and Computer Engineering, University of Minnesota, Minneapolis, MN, United States; ^7^ Center for Magnetic Resonance Research, University of Minnesota, Minneapolis, MN, United States

**Keywords:** T_RAFF2_ mapping, myocardial infarction, late gadolinium enhancement, T_1_, cardiovascular magnetic resonance, relaxation along a fictitious field

## Abstract

**Purpose:**

Evaluate the feasibility of quantification of Relaxation Along a Fictitious Field in the 2nd rotating frame (RAFF2) relaxation times in the human myocardium at 3 T.

**Methods:**

${\text{T}}_{\mathrm{RAFF}2}$ mapping was performed using a breath-held ECG-gated acquisition of five images: one without preparation, three preceded by RAFF2 trains of varying duration, and one preceded by a saturation prepulse. Pixel-wise ${\text{T}}_{\mathrm{RAFF}2}$ maps were obtained after three-parameter exponential fitting. The repeatability of ${\text{T}}_{\mathrm{RAFF}2}$, ${\text{T}}_{1}$, and ${\text{T}}_{2}$ was assessed in phantom via the coefficient of variation (CV) across three repetitions. In seven healthy subjects, ${\text{T}}_{\mathrm{RAFF}2}$ was tested for precision, reproducibility, inter-subject variability, and image quality (IQ) on a Likert scale (1 = Nondiagnostic, 5 = Excellent). Additionally, ${\text{T}}_{\mathrm{RAFF}2}$ mapping was performed in three patients with suspected cardiovascular disease, comparing it to late gadolinium enhancement (LGE), native ${\text{T}}_{1}$, ${\text{T}}_{2}$, and ECV mapping.

**Results:**

In phantom, ${\text{T}}_{\mathrm{RAFF}2}$ showed good repeatability (CV < 1.5%) while showing no ($R^{2}=0.09$) and high ($R^{2}=0.99$) correlation with ${\text{T}}_{1}$ and ${\text{T}}_{2}$, respectively. Myocardial ${\text{T}}_{\mathrm{RAFF}2}$ maps exhibited overall acceptable image quality (IQ = 3.0±1.0) with moderate artifact levels, stemming from off-resonances near the coronary sinus. Average ${\text{T}}_{\mathrm{RAFF}2}$ time across subjects and repetitions was 79.1 ± 7.3 ms. Good precision (7.6 ± 1.4%), reproducibility (1.0 ± 0.6%), and low inter-subject variability (10.0 ± 1.8%) were obtained. In patients, visual agreement of the infarcted area was observed in the ${\text{T}}_{\mathrm{RAFF}2}$ map and LGE.

**Conclusion:**

Myocardial ${\text{T}}_{\mathrm{RAFF}2}$ quantification at 3 T was successfully achieved in a single breath-hold with acceptable image quality, albeit with residual off-resonance artifacts. Nonetheless, preliminary clinical data indicate potential sensitivity of ${\text{T}}_{\mathrm{RAFF}2}$ mapping to myocardial infarction detection without the need for contrast agents, but off-resonance artifacts mitigation warrants further investigation.

## 1 Introduction

Late gadolinium enhancement (LGE) is the gold standard for detecting scar and replacement fibrosis after myocardial infarction (MI). Accumulated gadolinium-based contrast agents (GBCA) in LGE generate high contrast between healthy and infarcted myocardial regions, providing accurate infarct location, size, and viability information (1–3). LGE is also employed for differential diagnosis of non-ischemic cardiomyopathies, with patterns showing high predictive value (4–7). GBCA use in LGE is limited due to contraindications in acute and chronic renal insufficiency, risking nephrogenic systemic fibrosis (1, 8). Furthermore, adverse reactions to GBCA have been reported (9), as well as GBCA deposition in certain brain areas, particularly with repeated use (10). A major limitation of LGE is that the subjective qualitative images can only reveal hyperenhancement relative to normal reference tissue. This hampers inter-reader comparability and makes it difficult to detect diffuse fibrosis, which may affect the entire myocardium. In these cases, relative signal intensities in LGE images may fail to identify the disease burden. Furthermore, the relative signal intensity in LGE images is influenced by acquisition parameters, such as inversion time or slice thickness. During post-processing, intensity cut-offs in arbitrary units are typically used to distinguish normal myocardium from scar tissue and fibrosis. This approach leads to large inter-observer variability and a lack of reproducibility (11). Additionally, the qualitative signal intensities in LGE images cannot be compared across different scans or subjects. Finally, GBCAs accumulate in the extracellular matrix (ECM), which changes in various cardiac pathologies due to processes like inflammation, fibrosis, and altered vascular permeability (12). Therefore, GBCA are non-specific and typically cannot differentiate between these underlying pathophysiological processes. In contrast, endogenous MRI methods may accurately distinguish these biological processes in the ECM because they are directly sensitive to (macro)molecular interactions.

Quantitative cardiac MRI techniques, such as ${\text{T}}_{1}$ and ${\text{T}}_{2}$ (laboratory frame relaxation times) or ${\text{T}}_{1\rho }$ (rotating frame relaxation time) have been explored as non-contrast alternatives to LGE for MI detection. Native ${\text{T}}_{1}$ and ${\text{T}}_{2}$ mapping have shown promise in detecting MI (13–16), but its sensitivity and specificity remain the subject of debate (17, 18). In conventional ${\text{T}}_{1}$ and ${\text{T}}_{2}$ relaxation, relaxation occurs during free precession. ${\text{T}}_{1}$ relaxation is primarily influenced by interactions at the Larmor frequency (correlation times in $10^{-8}$ – $10^{-9}$ sec range), which, in clinical MRI, typically ranges from 10 to 100 MHz. ${\text{T}}_{2}$ relaxation, on the other hand, is sensitive to ultra-low-frequency interactions non-selectively.

To specifically target the intermediate frequency range, rotating frame relaxation times can be used (19). These occur during on-resonance radiofrequency (RF) irradiation, making the longitudinal [${\text{T}}_{1\rho }$ (20)] and transverse [${\text{T}}_{2\rho }$ (21)] rotating frame relaxation times sensitive to slow molecular processes with frequencies close to the RF pulse frequency, typically between 0.1 and 10 kHz in vivo. Conventional ${\text{T}}_{1\rho }$ maps are obtained using spin-lock (SL) preparation pulses of various durations, usually based on continuous-wave RF irradiation (22). Multiple studies have shown the sensitivity of ${\text{T}}_{1\rho }$ to MI and associated pathological alterations at 1.5 T (1, 8). Moreover, ${\text{T}}_{1\rho }$ relaxation has received increasing attention as an alternative imaging contrast with increased sensitivity to scar and fibrosis compared with native ${\text{T}}_{1}$ mapping. However, ${\text{T}}_{1\rho }$ relaxation using continuous-wave RF irradiation is sensitive to system imperfections (e.g., ${\text{B}}_{1}^{+}$ and ${\text{B}}_{0}$ inhomogeneities) (23, 24). To improve resilience against system imperfections, adiabatic SL pulses can be employed (25). During the adiabatic full passage frequency sweep, magnetization is locked along the effective field, making adiabatic ${\text{T}}_{1\rho }$ the dominant relaxation mechanism. Each adiabatic ${\text{T}}_{1\rho }$ preparation probes a wider spectrum of SL frequencies compared to the mono-frequency conventional SL, varying the effective field strength, orientation, and the angle between the effective field and the magnetization. A significant limitation of ${\text{T}}_{1\rho }$ relaxation time measurement is the relatively high specific absorption rate (SAR), which describes the energy absorbed into tissue, i.e. heating of the tissue during the imaging (18, 26). Thus, its applicability is limited in clinical settings, especially at high static magnetic field strengths (≥3 T).

Relaxation Along a Fictitious Field in the rotating frame of rank n (RAFFn) (27, 28) is an alternative rotating frame relaxation method with lower SAR requirements than ${\text{T}}_{1\rho }$ (29). RAFF2 has been shown to reduce SAR values by 30% compared to ${\text{T}}_{1\rho }$ measurements (28). RAFFn involves relaxation along a fictitious field in the nth rotating frame, created by nested sine amplitude- (AM) and cosine frequency-modulated (FM) RF pulses operating in a sub-adiabatic regime. This fast sub-adiabatic sweep of the effective RF field generates a fictitious field, which contributes to the final effective RF field, around which magnetization precesses (30). Like ${\text{T}}_{1\rho }$, RAFFn is selectively sensitive to dipolar interactions and slow microscopic molecular motions with fluctuation frequencies close to the rotating frame RF pulse amplitude (correlation times in $10^{-1}$ – $10^{-5}$ sec range). In vivo studies at 1.5 T (31, 32), as well as pre-clinical studies at 9.4 T in a mouse model (33, 34), have demonstrated sensitivity of relaxation times obtained with RAFF in the 2^nd^ rotating frame (${\text{T}}_{\mathrm{RAFF}2}$) to myocardial alterations in acute and chronic MI.

This study aimed to assess the feasibility of ${\text{T}}_{\mathrm{RAFF}2}$ mapping in the myocardium on a clinical 3 T scanner, where conventional SL imaging is greatly limited by SAR requirements and susceptibility to ${\text{B}}_{1}^{+}$ and ${\text{B}}_{0}$ inhomogeneities. A single breath-hold (BH) sequence using RAFF2 preparations is proposed. ${\text{T}}_{\mathrm{RAFF}2}$ quantification and repeatability are investigated in phantom and healthy subjects. Finally, clinical feasibility is evaluated in a small proof-of-principle cohort of patients with suspected cardiovascular disease.

## 2 Materials and methods

### 2.1 Pulse sequence design and reconstruction

As described by Liimatainen et al. (27), RAFF2 pulses consist of AM and FM RF pulses operating in a sub-adiabatic condition. These pulses are formulated to attain a stationary (constant and uniform) effective ($B_{\mathrm{eff}}(t)$) and fictitious field (F(t)) in the second frame of reference (doubly rotating frame). The AM and FM functions of RAFF pulses, based on *sine* and *cosine* of equal amplitude, are given by:

(1) $$\omega (t)=\omega_{1}^{\max}|\sin\;(\omega_{1}^{\max}t+\varphi_{0})|;$$

(2) $$\Delta \omega (t)=\omega_{1}^{\max}\cos\;(\omega_{1}^{\max}t+\varphi_{0}),\;$$

where $\omega_{1}^{\max}$ denotes the maximum pulse frequency in Equations 1, 2. The FM function as given in Equation 2 is converted to the phase modulation function:

(3) $$\phi (t)=\int_{0}^{t}\Delta \omega (t^{\prime })\;dt^{\prime }.$$

**Figure 1:**
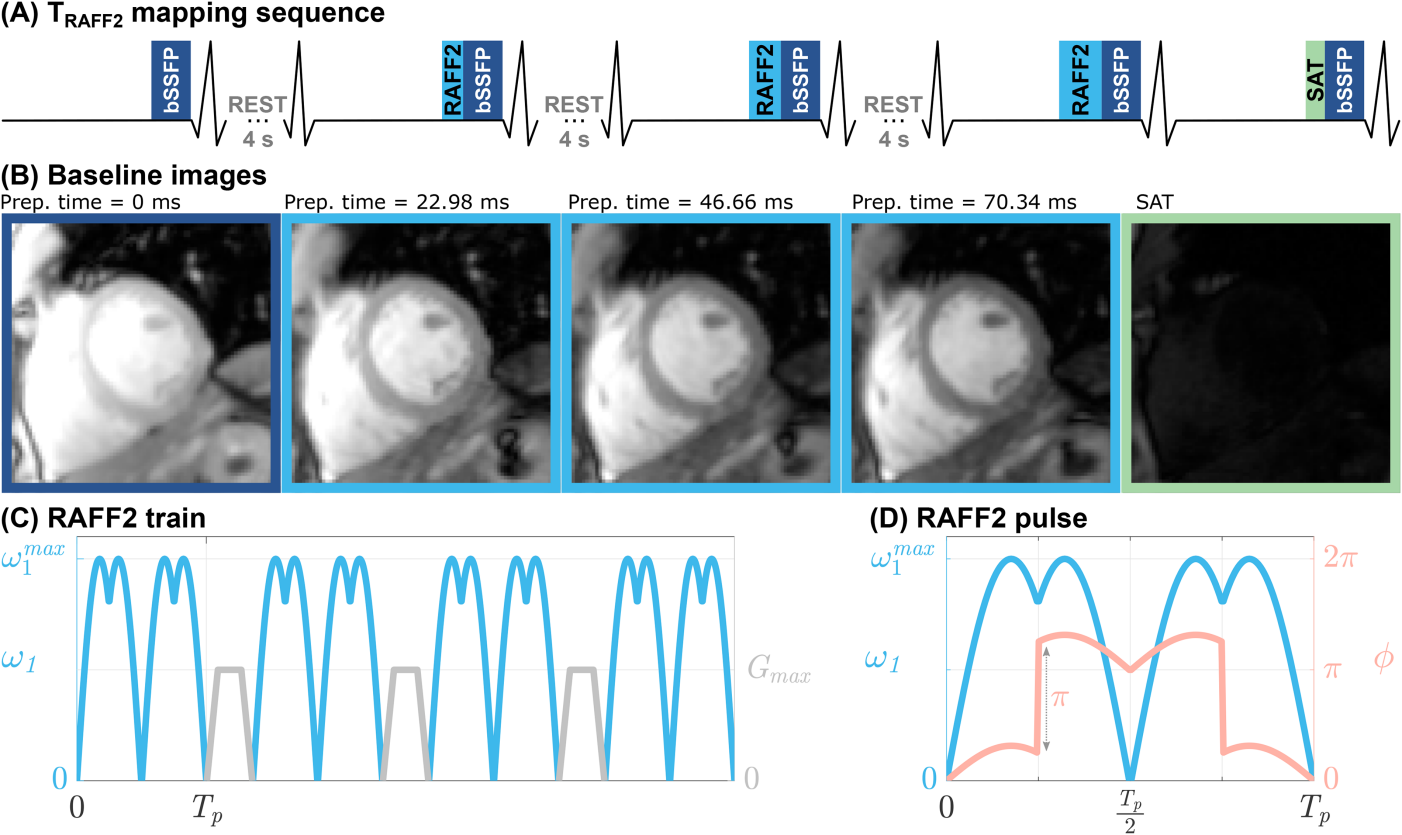
**(A)** Pulse sequence diagram for the proposed myocardial ${\text{T}}_{\mathrm{RAFF}2}$ mapping sequence. Five ECG-triggered single-shot balanced steady-state free precession (bSSFP) images **(B)** are acquired during end-diastole in a single breath-hold of ≈17 s. The first image is acquired with no preparation pulses to image the fully relaxed magnetization signal. Then, three images are acquired with different RAFF2-weightings, by concatenating a different number of RAFF2 pulses in a single preparation train. Finally, an image preceded by a saturation (SAT) pulse is acquired in the last heartbeat. Four-second rest periods are interleaved in the acquisition to allow for magnetization recovery. **(C)** Representative RAFF2 pulse train consisting of four RAFF2 pulses (blue) interleaved with randomized gradient spoiling (gray). **(D)** Amplitude (blue) and phase (pink) modulation function of a single RAFF2 radio-frequency (RF) pulse block, as described in Equations 1–3, where $\omega_{1}^{\max}$ is the RF pulse frequency and $T_{p}$ is the pulse duration.

The proposed ${\text{T}}_{\mathrm{RAFF}2}$ mapping sequence obtains five ECG-triggered single-shot images with various contrast weightings, acquired during end-diastole (Figures 1A, B). The first image was acquired without any magnetization preparation to enable sampling of the fully recovered magnetization. Subsequently, three RAFF2-weighted images were acquired after RAFF2 preparation blocks of varying duration (27). Each RAFF2 preparation block consisted of a varying number of RAFF2 pulses interleaved with randomized gradient spoiling to avoid imaging artifacts, as shown in the representative example of Figure 1C. To ensure full magnetization recovery, each RAFF2-weighted image was preceded by a four-second rest period. Finally, to capture the effect of imaging pulses on the magnetization recovery curve in a three-parameter fit model, an additional image was acquired directly after a saturation pulse (35), which mimicked the effect of a very long ${\text{T}}_{\mathrm{RAFF}2}$ preparation (∞ ms). Magnetization saturation was achieved with a “Water suppression Enhanced through ${\text{T}}_{1}$-effects” (WET) saturation module (36). Standard ECG gating with 4 electrodes was used, maintaining identical trigger delay times (time interval between the R-wave and the beginning of data acquisition) across images to ensure consistent cardiac cycle phases during breath-holding.

After data acquisition, ${\text{T}}_{\mathrm{RAFF}2}$ maps are generated by voxel-wise nonlinear least-squares curve-fitting to the magnitude signal intensity using a three-parameter model:

(4) $$S(T_{p})=A\cdot e^{-T_{p}/T_{\mathrm{RAFF}2}}+B.$$

Here $T_{p}$ is the duration of the RAFF2 preparation block. The three-parameter curve fitting model, as given in Equation 4, accounts for deviations in the magnetization curve resulting from the imaging pulses performed between the RAFF2 preparation block and the acquisition of the central k-space line. Parameters A and B depend on sequence parameters (such as flip angle, repetition time, number of pulses, etc.) and remain unaffected by the duration of the RAFF2 preparation blocks. Additionally, parameter B specifically accounts for the impact of imaging pulses when the longitudinal magnetization reaches zero. Spatially-resolved standard deviation (SD) maps were obtained from the fit residuals as an estimate of the ${\text{T}}_{\mathrm{RAFF}2}$ precision (37).

### 2.2 MR imaging

The proposed ${\text{T}}_{\mathrm{RAFF}2}$ mapping sequence was evaluated on a 3 T MRI scanner (Magnetom Prisma; Siemens Healthineers, Erlangen, Germany) using a body coil for transmission and a 24-channel receiver coil array.

Typical preparation parameters for the ${\text{T}}_{\mathrm{RAFF}2}$ mapping were RF pulse frequency = 625 Hz (pulse duration = 2.26 ms), total prep times (including spoiling duration) = 22.98, 46.66, 70.34 ms (number of RAFF2 pulse repetitions = 8, 16, 24), and gradient spoiler duration =  0.7 ms. The remaining imaging parameters were: field-of-view (FOV) = 340 × 270 ${\text{mm}}^{2}$, in-plane resolution = 1.8 × 1.8 ${\text{mm}}^{2}$, slice thickness = 8 mm, partial Fourier factor = 6/8, readout type = balanced steady-state free precession (bSSFP), flip angle (FA) = $70^{\circ }$, segments = 73, bandwidth = 1185 Hz/px, GRAPPA factor/reference lines = 2/24, TR/TE = 3.0/1.2 ms, k-space ordering = linear, and scan time ≈17 s.

For reference, single-slice ${\text{T}}_{1}$ mapping was acquired, consisting of a 5-(3s)-3 modified Look-Locker inversion recovery (MOLLI) (38) sequence with similar imaging parameters except for FA = $35^{\circ }$, TR/TE = 2.5/1.0 ms, and scan time ≈11 s. Single-slice ${\text{T}}_{2}$ mapping was obtained using a ${\text{T}}_{2}$-prepared bSSFP with ${\text{T}}_{2}$ prep duration of 0, 25, and 50 ms (35).

#### 2.2.1 Phantom

For the phantom experiments, the T1MES phantom (39), consisting of nine vials with different concentrations of agarose gel and nickel chloride, was used. Reference measurements for ${\text{T}}_{\mathrm{RAFF}2}$ were performed using an gradient echo sequence with the same preparation parameters and imaging parameters as the proposed ${\text{T}}_{\mathrm{RAFF}2}$ mapping, except TR/TE = 10 000/3.26 ms, FA = $90^{\circ }$, no GRAPPA, and 1 k-space line per readout. ${\text{T}}_{\mathrm{RAFF}2}$, ${\text{T}}_{1}$, and ${\text{T}}_{2}$ mapping were performed with in-line fitting and map generation. The average (± SD) ${\text{T}}_{\mathrm{RAFF}2}$, ${\text{T}}_{1}$, and ${\text{T}}_{2}$ times were obtained by manually drawing a circular region of interest (ROI) for each vial. ${\text{T}}_{\mathrm{RAFF}2}$ was compared with ${\text{T}}_{1}$ and ${\text{T}}_{2}$ using Pearson’s linear correlation coefficient. To assess the intra-scanner repeatability of ${\text{T}}_{\mathrm{RAFF}2}$, ${\text{T}}_{1}$, and ${\text{T}}_{2}$ mapping, three repetitions of the same imaging slice (without repositioning) were performed in a single scanning session and the coefficient of variation (CV) was computed for each vial.

Further, the performance of ${\text{T}}_{\mathrm{RAFF}2}$ mapping in the presence of ${\text{B}}_{0}$ and ${\text{B}}_{1}^{+}$ inhomogeneity was evaluated in two separate experiments. ${\text{T}}_{\mathrm{RAFF}2}$ maps were acquired across a range of ${\text{B}}_{0}$ off-resonances ($\Delta \omega_{0}=[-150,\;-100,\;-50,\;-25,\;0,\;25,\;50,\;100,\;150]$ Hz) and relative ${\text{B}}_{1}^{+}$ strengths (scaling factor $\eta_{1}$ = [0.4, 0.6, 0.8, 1.0]) by modifying the frequency offset and RAFF pulse strength, respectively. Additionally, ${\text{B}}_{0}$ (40) and ${\text{B}}_{1}^{+}$ (41) mapping was performed at the original center frequency ($\Delta \omega_{0}$ = 0). The ${\text{B}}_{0}$ and ${\text{B}}_{1}^{+}$ resilience of ${\text{T}}_{\mathrm{RAFF}2}$ measurements was evaluated in three vials with ${\text{T}}_{1}$ and ${\text{T}}_{2}$ times corresponding to those of native myocardial tissue, post-contrast myocardial tissue, and native blood, respectively (39). For each vial and each value of $\Delta \omega_{0}$ or $\eta_{1}$, the deviation of ${\text{T}}_{\mathrm{RAFF}2}$ times was calculated relative to the ${\text{T}}_{\mathrm{RAFF}2}$ values on-resonance ($\Delta \omega_{0}$ = 0) or at the original pulse amplitude ($\eta_{1}$ = 1.0), respectively.

#### 2.2.2 In vivo

Myocardial ${\text{T}}_{\mathrm{RAFF}2}$, ${\text{T}}_{1}$, and ${\text{T}}_{2}$ mapping were obtained in seven healthy subjects with no known cardiovascular disease history or contraindications to MRI (6 males; 35.4 ± 3.6 years), after obtaining written informed consent approved by the relevant institutional review board.

Single-slice ${\text{T}}_{\mathrm{RAFF}2}$ mapping was performed during a single (≈17 s) breath-hold (BH). Three short-axis (SAX) slices (basal, mid-ventricular, and apical) were acquired, and each slice was repeated three times. SAR burden (≈1.7 ± 0.1 W/kg) was kept below the standard operation threshold (whole-body SAR < 2.0 W/kg) and no first-level mode was enabled.

Additionally, single-slice (mid-SAX) ${\text{T}}_{1}$ and ${\text{T}}_{2}$ mapping was acquired.

To reduce residual in-plane motion, image registration was applied to the baseline images using a group-wise registration method based on principal component analysis (42). Myocardial segmentation was performed using a nnU-Net framework with Bayesian uncertainty estimation (43), and segmentation maps with predictive confidence below 75% were manually revised. The average (±SD) values of ${\text{T}}_{\mathrm{RAFF}2}$ in the segmented myocardium were extracted according to the American Heart Association (AHA) 16-segment model (44).

A group-wise ANOVA test followed by paired t-tests was used to assess statistical differences between the ${\text{T}}_{\mathrm{RAFF}2}$ times for each slice. A p-value < 0.05 was considered statistically significant. For each myocardial segment, the precision (wCV), reproducibility ($\bar{wCV}$), and inter-subject variability ($\bar{CV}$) of ${\text{T}}_{\mathrm{RAFF}2}$ were assessed as previously defined (45).

The ${\text{T}}_{\mathrm{RAFF}2}$, ${\text{T}}_{1}$, and ${\text{T}}_{2}$ maps were assessed using a 5-point Likert scale in terms of image quality/artifact level (1 = Nondiagnostic/Nondiagnostic, 2 = Poor/Severe, 3 = Acceptable/Moderate, 4 = Good/Mild, 5 = Excellent/Minimal) by two independent readers with over 10 years of imaging experience. Statistical differences between the image quality scores of each parametric map were investigated using a group-wise Kruskal–Wallis test and subsequently Mann–Whitney U-tests.

Three patients (3 males, 63.0 ± 8.3 years) with suspected or known cardiac diseases were recruited for cardiac magnetic resonance (CMR) imaging. Mid-SAX or 4-chamber view (4CH) pre-contrast (native) ${\text{T}}_{\mathrm{RAFF}2}$, ${\text{T}}_{1}$, and ${\text{T}}_{2}$ mapping as well as post-contrast ${\text{T}}_{1}$ mapping and phase-sensitive inversion recovery (PSIR) LGE (46) were acquired. Extracellular volume (ECV) maps were estimated from pre-contrast and post-contrast ${\text{T}}_{1}$ values.

LGE imaging was performed no longer than 10 min after injection of 0.1 mmol/kg gadoterate meglumine (Dotarem, Guerbet, Villepinte, France) contrast agent with the following parameters: FOV = 430 × 322 ${\text{mm}}^{2}$, in-plane resolution = 1.7 × 1.7 ${\text{mm}}^{2}$, slice thickness = 8 mm, FA = $50^{\circ }$, segments = 72, GRAPPA factor/reference lines = 2/32, TR/TE = 2.2/1.1 ms. To maintain the same matrix size as in the healthy subject imaging, ${\text{T}}_{\mathrm{RAFF}2}$ mapping was acquired with a lower in-plane resolution of 2.08 × 2.08 ${\text{mm}}^{2}$. ${\text{T}}_{1}$ and ${\text{T}}_{2}$ mapping used identical imaging parameters as in the healthy subject imaging.

Manually drawn ROIs were defined on LGE images and then superimposed on the co-registered quantitative maps to extract scar and remote ${\text{T}}_{\mathrm{RAFF}2}$, ECV, ${\text{T}}_{1}$, and ${\text{T}}_{2}$ times. Abnormal areas were determined as regions with hyperenhancement in the LGE images, using the two standard deviations (2SD) segmentation method (47). Here, remote areas were selected as regions with no visible hyperenhancement. All baseline images were co-registered with each other and their mean was computed (42). This mean image was then registered with the LGE image, and the motion fields were computed. These fields were subsequently applied to the ${\text{T}}_{\mathrm{RAFF}2}$, ECV, ${\text{T}}_{1}$, and ${\text{T}}_{2}$ maps.

## 3 Results

### 3.1 Phantom

**Figure 2:**
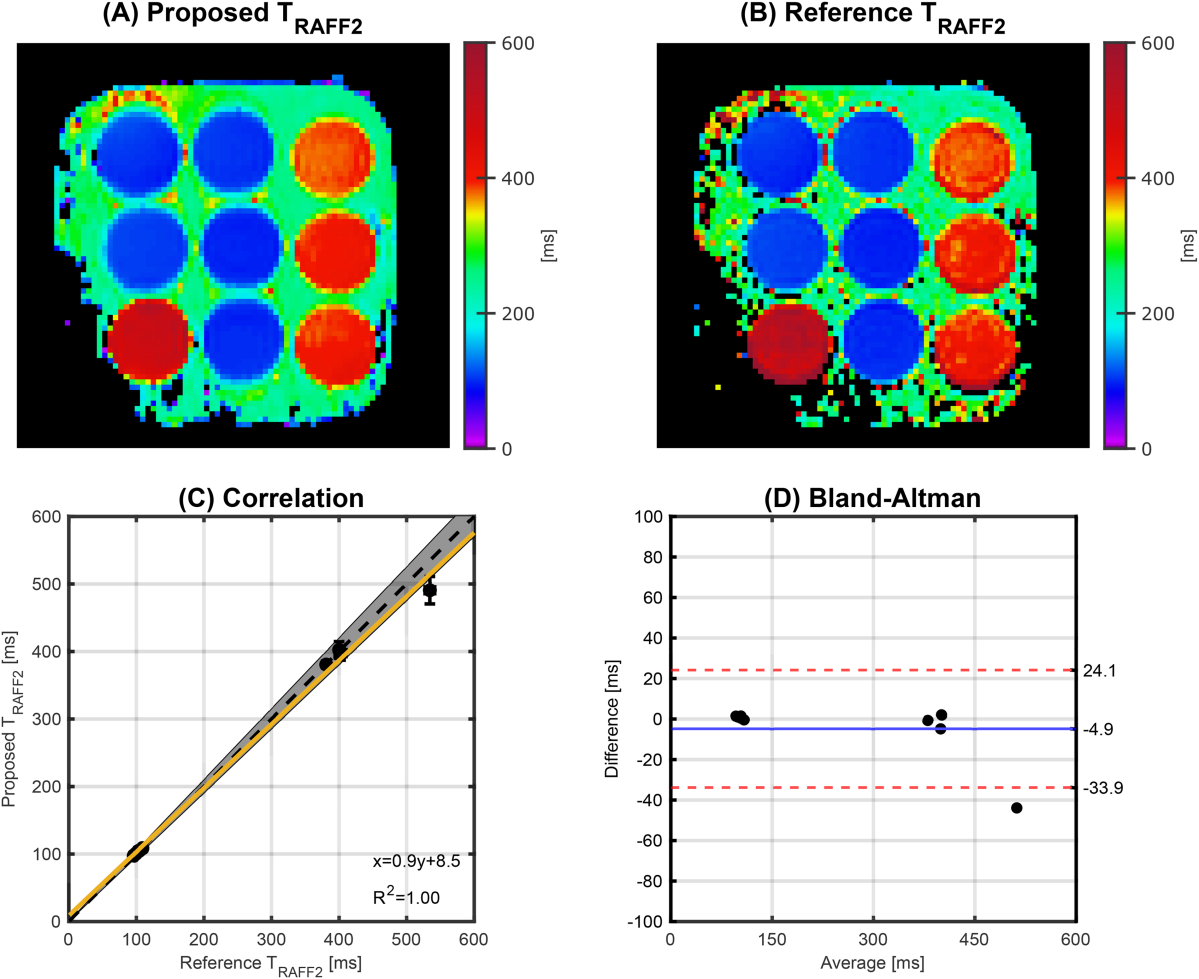
Phantom imaging using the **(A)** proposed and **(B)** reference ${\text{T}}_{\mathrm{RAFF}2}$ mapping technique. For all the nine vials, **(C)** correlation and **(D)** Bland-Altman plots are also displayed. The yellow line represents the best linear fit and the gray shading indicates a 5% deviation from the reference (black dashed line). The coefficient of determination ($R^{2}$) and the equation of the best linear fit are shown in the bottom right. In the Bland-Altman plot, the solid blue line represents the mean bias, and the red dashed lines represent the limits of agreement (±1.96 SD).

**Figure 3:**
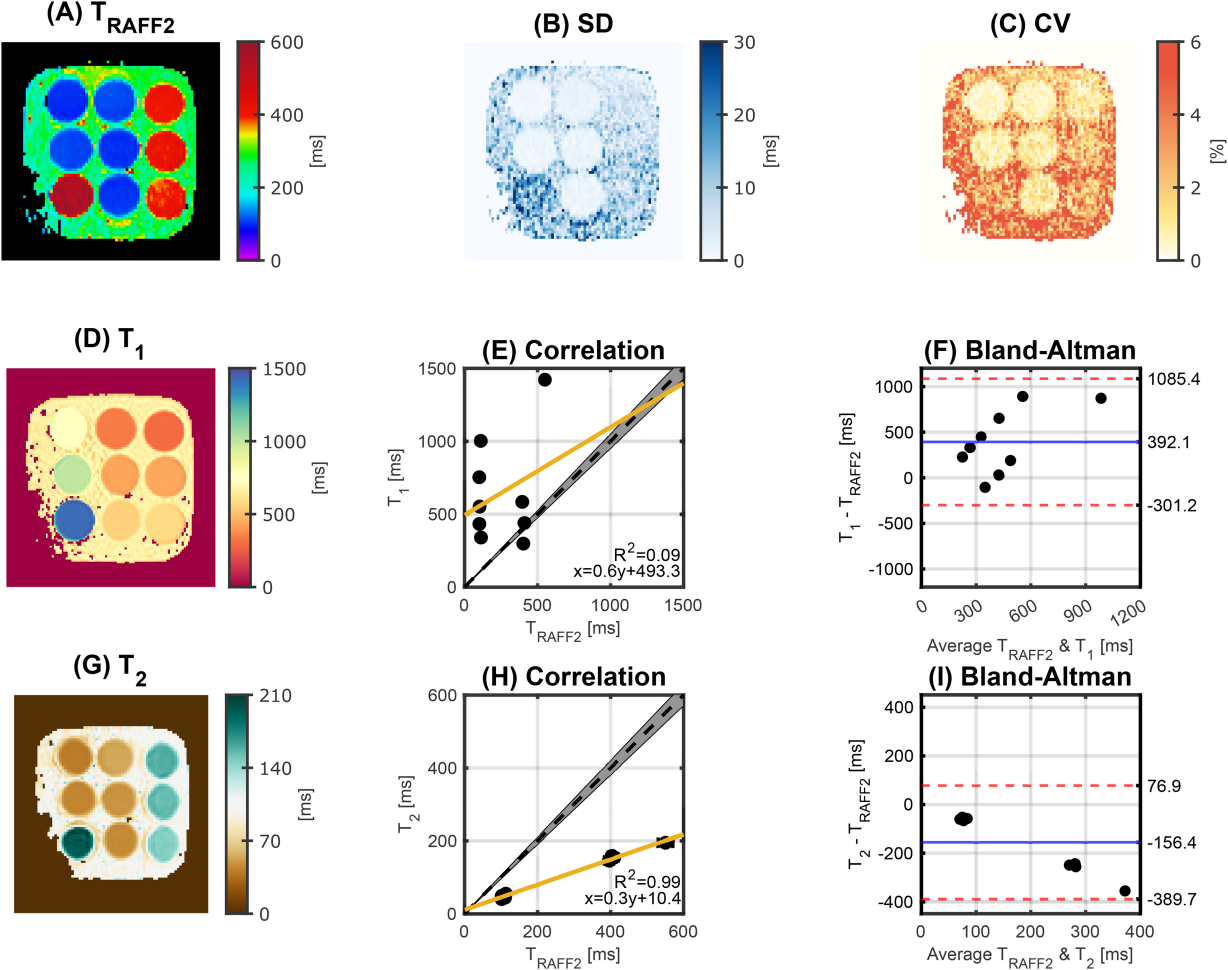
**(A)** ${\text{T}}_{\mathrm{RAFF}2}$ map and **(B)** corresponding standard deviation (SD) map obtained in phantom. **(C)** Coefficient of variation (CV) map of ${\text{T}}_{\mathrm{RAFF}2}$ across three repetitions. Excellent intra-scanner repeatability was achieved with a CV <3% for all the vials. **(D)** ${\text{T}}_{1}$ and **(G)** ${\text{T}}_{2}$ map. Correlation plot between ${\text{T}}_{\mathrm{RAFF}2}$ and **(E)** ${\text{T}}_{1}$ and **(H)** ${\text{T}}_{2}$. The yellow line represents the best linear fit and the gray shading indicates a 5% deviation from the reference (black dashed line). The coefficient of determination ($R^{2}$) and the equation of the best linear fit are shown in the bottom right. (**F**,**I**) Corresponding Bland-Altman plot. The solid blue line represents the mean bias, and the red dashed lines represent the limits of agreement (±1.96 SD). The concentration of agarose (%) / nickel chloride (mM) per vial (top, middle, and bottom row) is 2.3/0.9, 2.3/0.3, 0.4/0.2 (left column), 2.2/4.5, 2.3/2.9, 2.4/2.1 (middle column), and 0.2/5.6, 0.4/0.4, 0.3/2.9 (right column).

An excellent agreement was achieved between the proposed and the reference ${\text{T}}_{\mathrm{RAFF}2}$ mapping techniques ($R^{2}=1.00$), as shown in Figure 2. Phantom ${\text{T}}_{\mathrm{RAFF}2}$ and the corresponding SD map are shown in Figures 3A, B, respectively. ${\text{T}}_{\mathrm{RAFF}2}$ shows good sensitivity to changes in phantom composition with a range of 101.7±1.0−550.8±14.9 ms across the nine vials. Excellent precision (spatially-resolved SD obtained from the fit residuals) was measured with an average of 4.9 ± 2.3 ms. Excellent intra-scanner repeatability was obtained across the three repetitions with an average CV of 1.4 ± 0.7% (range: 0.5%–2.3%) for ${\text{T}}_{\mathrm{RAFF}2}$ (Figure 3C). The average CV for ${\text{T}}_{1}$ and ${\text{T}}_{2}$ was 0.4 ± 0.2% (0.3%–0.6%) and 0.5 ± 0.3% (0.3%–0.8%), respectively. Between ${\text{T}}_{\mathrm{RAFF}2}$ and ${\text{T}}_{1}$ (${\text{T}}_{1}$ range: 297.8±1.3−1421.6±5.7 ms, Figure 3D) no correlation was observed (${\text{R}}^{2}=0.09$), as shown in Figures 3E,F. Between ${\text{T}}_{\mathrm{RAFF}2}$ and ${\text{T}}_{2}$, a high correlation ($R^{2}=0.99$) with a bias of −156.4 ms was observed (${\text{T}}_{2}$ range: 40.1±0.4−194.4±3.4 ms), as shown in Figures 3G–I. Excellent agreement was observed for ${\text{T}}_{1}$ ($R^{2}=0.99$) and ${\text{T}}_{2}$ ($R^{2}=0.96$) when compared with the literature values (39).

**Figure 4:**
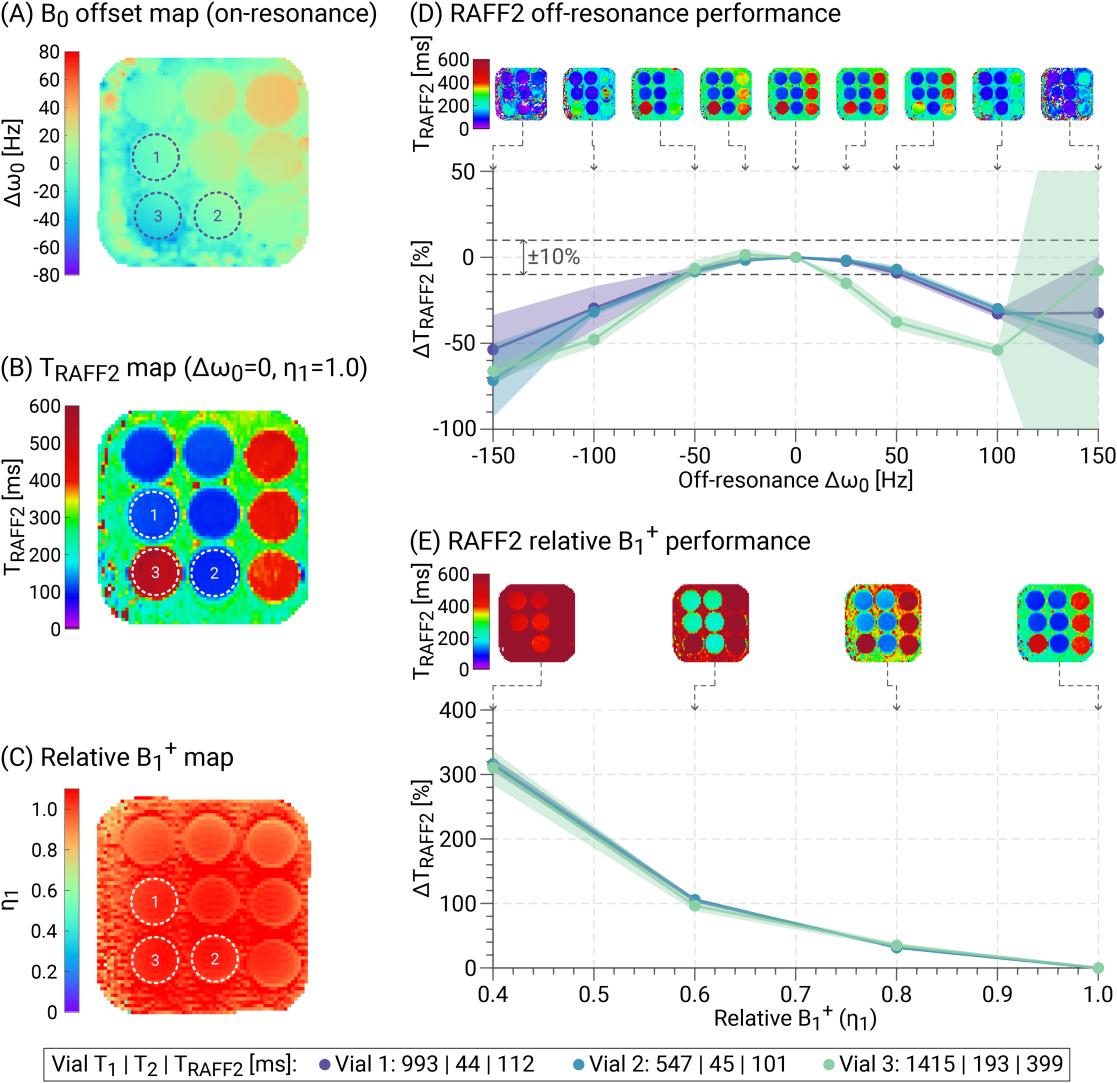
${\text{B}}_{0}$ and ${\text{B}}_{1}^{+}$ resilience of ${\text{T}}_{\mathrm{RAFF}2}$ measurements in phantom. **(A)** ${\text{B}}_{0}$ frequency offset ($\Delta \omega_{0}$) and **(C)** relative ${\text{B}}_{1}^{+}$ strength ($\eta_{1}$) maps. **(B)** ${\text{T}}_{\mathrm{RAFF}2}$ map acquired on-resonance ($\Delta \omega_{0}$ = 0) with original pulse amplitude ($\eta_{1}$ = 1.0). ${\text{T}}_{\mathrm{RAFF}2}$ values were evaluated in three vials with ${\text{T}}_{1}$/${\text{T}}_{2}$ times corresponding to native myocardium (vial 1), post-contrast myocardium (vial 2), and native blood (vial 3) (39), across different levels of **(D)** off-resonance and **(E)** relative ${\text{B}}_{1}^{+}$ strength. **(D)** Increasing off-resonance led to an underestimation of ${\text{T}}_{\mathrm{RAFF}2}$ compared with the on-resonance value ($\Delta \omega_{0}$ = 0). The deviations remained below 10% within a window of ±50 Hz for vials 1 and 2, and within ±35 Hz for vial 3. **(E)** As $\eta_{1}$ decreased, ${\text{T}}_{\mathrm{RAFF}2}$ was progressively overestimated relative to the value at original pulse amplitude ($\eta_{1}$ = 1.0). Given the observed levels of ${\text{B}}_{0}$ (−9.3 ± 11.5 Hz) and ${\text{B}}_{1}^{+}$ inhomogeneity ($\eta_{1}$ = 1.05 ± 0.01) across the three vials, the resulting bias in ${\text{T}}_{\mathrm{RAFF}2}$ was estimated to be well below 5% in phantom.

The performance of ${\text{T}}_{\mathrm{RAFF}2}$ mapping in the presence of ${\text{B}}_{0}$ and ${\text{B}}_{1}^{+}$ changes is illustrated in Figure 4. ${\text{T}}_{\mathrm{RAFF}2}$ times were progressively underestimated with increasing off-resonance values ($\left|\Delta \omega_{0}\right|$), consistent with previous findings (48). For vial 1 (${\text{T}}_{\mathrm{RAFF}2}$ = 112 ± 2 ms) and vial 2 (${\text{T}}_{\mathrm{RAFF}2}$ = 101 ± 2 ms), the deviations remained below 10% within an off-resonance window of ±50 Hz, and for vial 3 (${\text{T}}_{\mathrm{RAFF}2}$ = 399 ± 19 ms), within a window of ±35 Hz. At the original center frequency, i.e. without added offset ($\Delta \omega_{0}$ = 0), the average ${\text{B}}_{0}$ off-resonance was −9.3 ± 11.5 Hz across the three vials, resulting in an estimated bias in ${\text{T}}_{\mathrm{RAFF}2}$ times of approximately 2%. As the pulse amplitude decreased, ${\text{T}}_{\mathrm{RAFF}2}$ times increased over the range of relative ${\text{B}}_{1}^{+}$ scaling factors up to 314 ± 3% at $\eta_{1}$ = 0.4. This is in line with previously reported results (48). The relative ${\text{B}}_{1}^{+}$ strength across the selected vials was 1.05 ± 0.01.

### 3.2 In vivo

**Figure 5:**
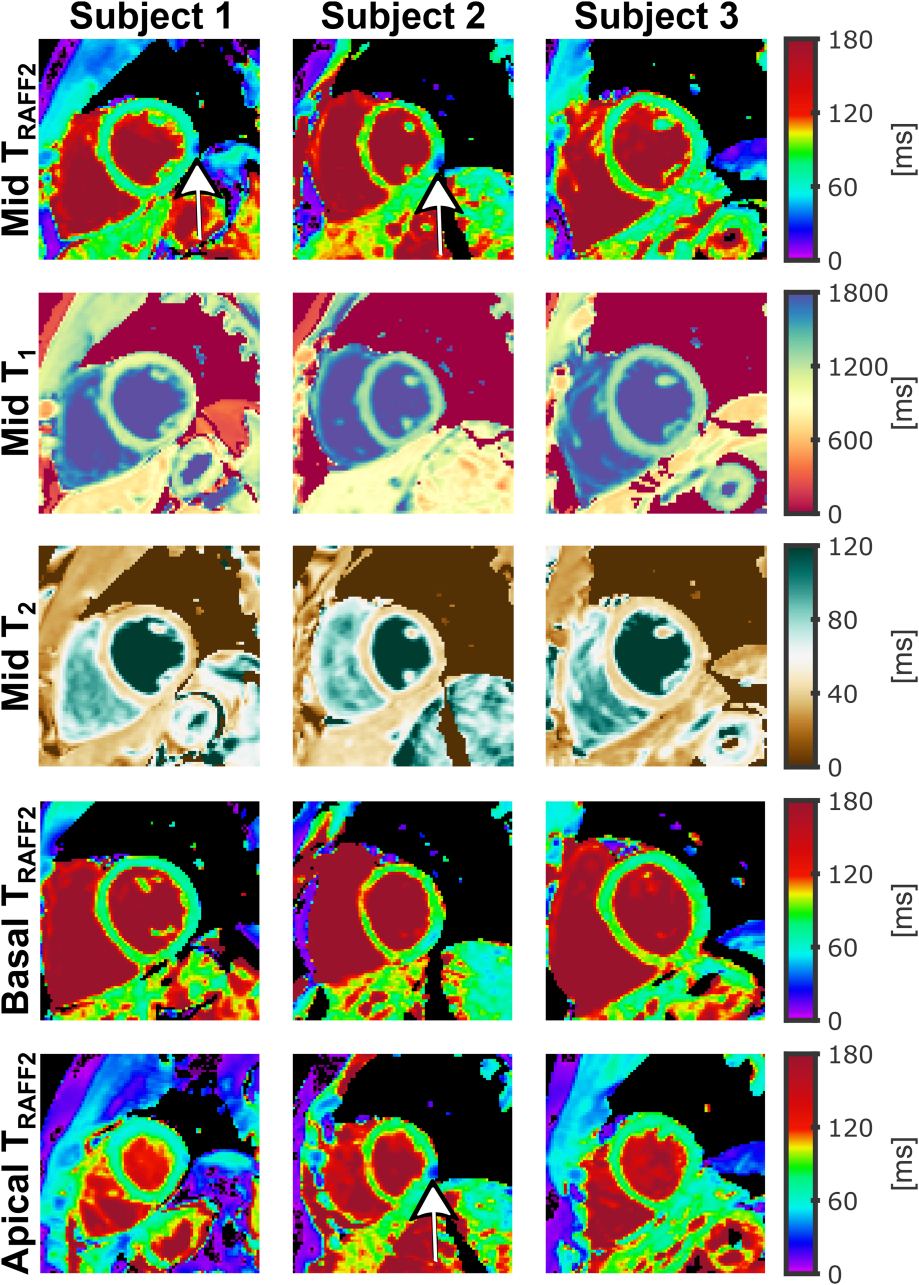
${\text{T}}_{\mathrm{RAFF}2}$, ${\text{T}}_{1}$, and ${\text{T}}_{2}$ maps acquired for three different healthy subjects. ${\text{T}}_{\mathrm{RAFF}2}$ was acquired for mid-ventricular (first row), basal (fourth row), and apical (fifth row) short-axis (SAX) slices. ${\text{T}}_{1}$ (second row) and ${\text{T}}_{2}$ (third row) were acquired for a single mid-ventricular SAX slice without repositioning. Visually homogeneous ${\text{T}}_{\mathrm{RAFF}2}$ maps were obtained in these subjects with similar values across slices and subjects, despite residual ${\text{B}}_{0}$-related (off-resonance) artifacts being visible in some images (white arrows).

Figure 5 shows ${\text{T}}_{\mathrm{RAFF}2}$ maps in three SAX slices as well as single mid-SAX ${\text{T}}_{1}$ and ${\text{T}}_{2}$ maps acquired in three representative healthy subjects. In vivo myocardial ${\text{T}}_{\mathrm{RAFF}2}$ maps were obtained with acceptable visual map quality and low variability throughout the myocardium, with a fairly homogeneous depiction of the myocardium for the three SAX slices. Residual artifacts are visually apparent in some ${\text{T}}_{\mathrm{RAFF}2}$ maps, particularly in areas of strong off-resonance (e.g., around the coronary sinus).

**Figure 6:**
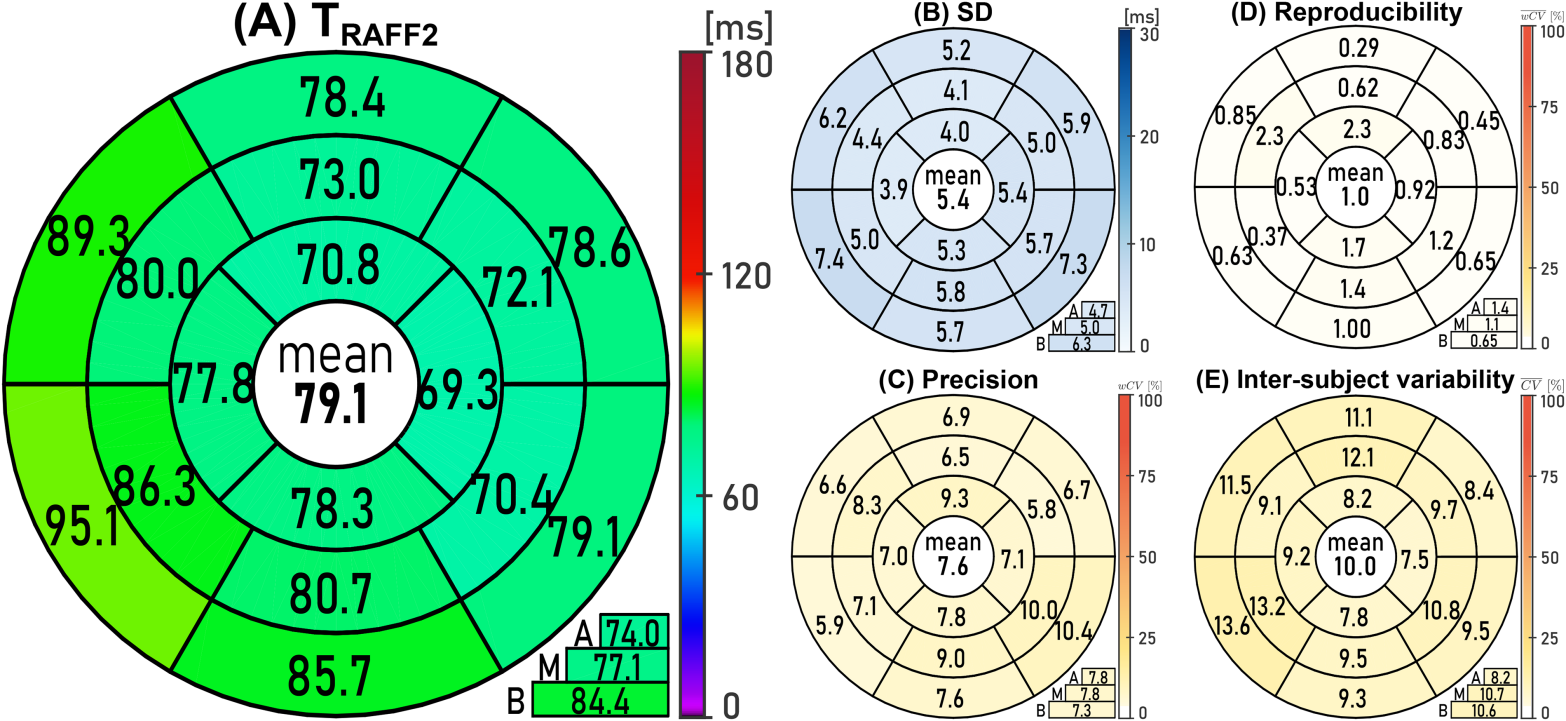
Bullseye plots with the American Heart Association (AHA) myocardial 16-segments containing the average **(A)** ${\text{T}}_{\mathrm{RAFF}2}$ and **(B)** standard deviation (SD) across all healthy subjects and repetitions, as well as the average **(C)** precision (wCV), **(D)** reproducibility ($\bar{wCV}$), and **(E)** inter-subject variability ($\bar{CV}$) coefficients for ${\text{T}}_{\mathrm{RAFF}2}$ mapping. Global average values are reported at the center of each bullseye plot and the average of the three short-axis slices (A, apical; M, mid-ventricular; B, basal) is shown in the lower right.

Figure 6A shows the 16-segment bullseye plot with the average ${\text{T}}_{\mathrm{RAFF}2}$ values across all healthy subjects with an average ${\text{T}}_{\mathrm{RAFF}2}$ value of 79.1 ± 7.3 ms. Notably, a slight trend of increased times in the septum was observed, with average ${\text{T}}_{\mathrm{RAFF}2}$ values of 74.0 ± 4.7 ms, 77.1 ± 6.2 ms, and 84.4 ± 6.9 ms for the apical, mid-ventricular, and basal slices, respectively. In visual inspection, this trend can be linked to the aforementioned off-resonance artifact. Additionally, the lower ${\text{T}}_{\mathrm{RAFF}2}$ values in the apical slice may be explained with the higher contribution of ${\text{B}}_{0}$ inhomogeneities at the apex. The apical and basal ${\text{T}}_{\mathrm{RAFF}2}$ values differed significantly (*p* = 0.03), while there were no significant differences between mid-ventricular and basal (*p* = 0.08), and between mid-ventricular and apical (*p* = 0.43) ${\text{T}}_{\mathrm{RAFF}2}$ values. ${\text{T}}_{\mathrm{RAFF}2}$ mapping exhibited good precision across all healthy subjects with an average SD of 5.4 ± 1.0 ms (4.7 ± 0.8 ms, 5.0 ± 0.7 ms, and 6.3 ± 0.9 ms for the apical, mid-ventricular, and basal slices, respectively) and an average wCV of 7.6 ± 1.4% (7.8 ± 1.1%, 7.8 ± 1.6%, and 7.3 ± 1.6% for the apical, mid-ventricular, and basal slices, respectively), as shown in Figures 6B,C respectively.

Additionally, excellent reproducibility was obtained with an average $\bar{wCV}$ of 1.0 ± 0.6% (Figure 6D). The apical, mid-ventricular, and basal slices displayed $\bar{wCV}$ values of 1.4 ± 0.8%, 1.1 ± 0.7%, and 0.6 ± 0.3%, respectively. Furthermore, low inter-subject variability was observed with an average $\bar{CV}$ of 10.0 ± 1.8% (Figure 6E). The apical, mid-ventricular, and basal slices showed $\bar{CV}$ values of 8.2 ± 0.7%, 10.7 ± 1.6%, and 10.6 ± 1.9%, respectively.

The average score of the ${\text{T}}_{\mathrm{RAFF}2}$ maps was 3.0 ± 1.0 (3.2 ± 1.0, 3.0 ± 0.9, and 2.8 ± 1.0 for the apical, mid-ventricular, and basal slices, respectively), indicating acceptable image quality with a moderate level of artifacts. The ${\text{T}}_{1}$ and ${\text{T}}_{2}$ maps achieved a score of 3.8 ± 0.8 and 3.8 ± 0.9, respectively, indicating good image quality with a mild level of artifacts. While no statistical difference was found between the image scores of the ${\text{T}}_{\mathrm{RAFF}2}$ maps from different slices, the image score of the ${\text{T}}_{1}$ and ${\text{T}}_{2}$ maps was significantly higher than the ${\text{T}}_{\mathrm{RAFF}2}$ image score of any slice. Supplementary Figure S1 contains a representative example of ${\text{T}}_{\mathrm{RAFF}2}$ and ${\text{T}}_{1}$ maps for each image quality score.

**Figure 7:**
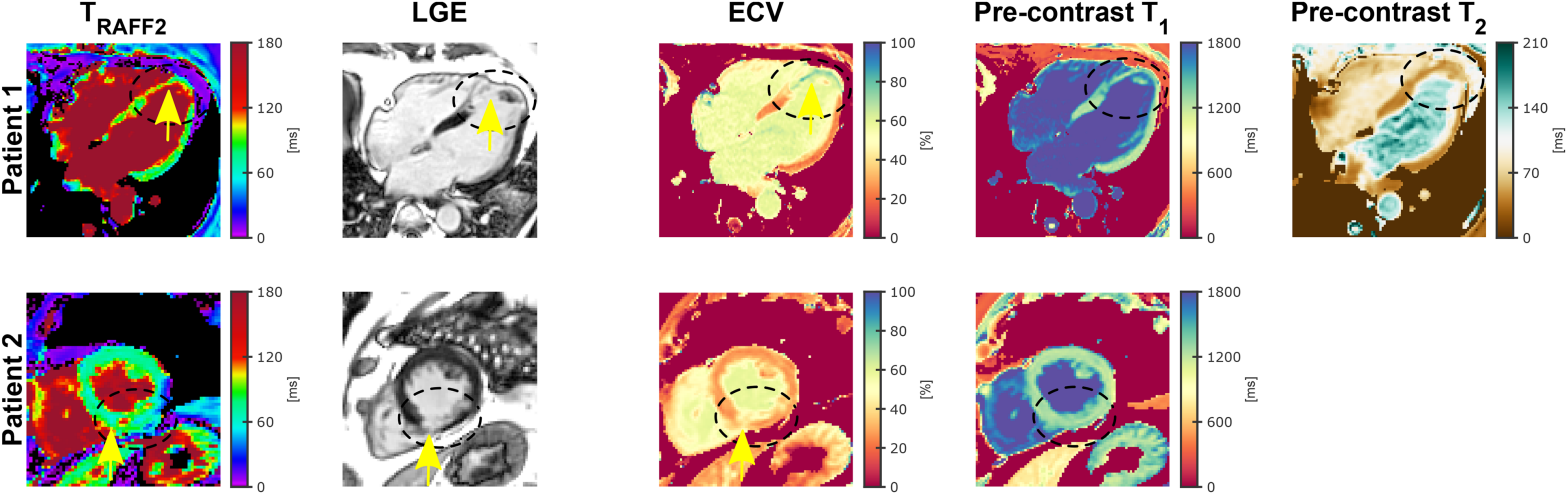
Native (pre-contrast) ${\text{T}}_{\mathrm{RAFF}2}$, late gadolinium enhancement (LGE), extracellular volume (ECV) map, and native ${\text{T}}_{1}$ and ${\text{T}}_{2}$ for two LGE-positive patients. (Top row) Fifty-two-year-old patient diagnosed with left anterior descending coronary artery (LAD) territory infarct with a left-ventricle (LV) apical thrombus. The black dashed ellipse represent the LGE-positive area, which shows an increase in infarct (yellow arrow) compared with remote areas for ${\text{T}}_{\mathrm{RAFF}2}$ and ECV. Pre-contrast ${\text{T}}_{1}$ and ${\text{T}}_{2}$ maps show no abnormality. (Bottom row) Sixty-five-year-old patient diagnosed with mid right coronary artery (RCA) infarct. Scar tissue (yellow arrow) in the inferior segment (black dashed ellipse) is visible in the ${\text{T}}_{\mathrm{RAFF}2}$, LGE, and ECV map while the native ${\text{T}}_{1}$ map shows no abnormality. No ${\text{T}}_{2}$ mapping was obtained for Patient 2 due to scanning time constraints.

Two out of three patients exhibited LGE-positive findings in the CMR examination, and the imaging slice intersected with the specific region of scar tissue identified in the LGE images. Figure 7 depicts the ${\text{T}}_{\mathrm{RAFF}2}$ maps alongside the corresponding LGE images, ECV, native ${\text{T}}_{1}$, and ${\text{T}}_{2}$ maps for the two patients with an infarct identified through LGE. No ${\text{T}}_{2}$ mapping was obtained for Patient 2 due to scanning time constraints. Mean remote and infarct ${\text{T}}_{\mathrm{RAFF}2}$, ECV, ${\text{T}}_{1}$, and ${\text{T}}_{2}$ values for the LGE-positive patients are reported in Table 1.

**Table 1:** Mean remote and infarct ${\text{T}}_{\mathrm{RAFF}2}$, extracellular volume (ECV), ${\text{T}}_{1}$, and ${\text{T}}_{2}$ values for the LGE-positive patients.

	Patient 1		Patient 2	
	Remote	Infarct	Remote	Infarct
${\text{T}}_{\mathrm{RAFF}2}$ [ms]	88.1 ± 11.7	92.6 ± 15.0	69.6 ± 2.3	87.4 ± 22.9
ECV [%]	22.8 ± 2.2	71.5 ± 3.3	25.9 ± 0.9	42.7 ± 4.6
${\text{T}}_{1}$ [ms]	1210.3 ± 26.2	1335.8 ± 125.5	1248.1 ± 97.2	1351.5±82.6
${\text{T}}_{2}$ [ms]	48.6 ± 7.6	49.4 ± 4.1	–	–

## 4 Discussion

In this study, the feasibility of myocardial ${\text{T}}_{\mathrm{RAFF}2}$ mapping was demonstrated in vivo at 3 T in a single breath-hold. Phantom experiments showed repeatable and noise-resilient ${\text{T}}_{\mathrm{RAFF}2}$ quantification. Promising results with largely homogeneous and reproducible ${\text{T}}_{\mathrm{RAFF}2}$ times were demonstrated in healthy subjects. Residual off-resonance artifacts were still observed in some maps leading to reduced map quality compared with ${\text{T}}_{1}$ mapping. Initial clinical data showed feasibility in patients and visual alignment of areas with altered ${\text{T}}_{\mathrm{RAFF}2}$ and hyperenhancement in LGE images may indicate potential sensitivity to myocardial injury.

Previous studies have shown early evidence of ${\text{T}}_{\mathrm{RAFF}2}$ sensitivity to myocardial injury at 1.5 T (31, 32). Based on this, the use of RAFF2 as a spin-locking mechanism for myocardial tissue characterization was evaluated at 3 T. CMR at 3 T can benefit from an increased intrinsic signal-to-noise ratio and is commonly used in expert centers. Due to the exacerbated restrictions imposed by the SAR limitations and the high sensitivity to ${\text{B}}_{1}^{+}$ and ${\text{B}}_{0}$ inhomogeneities, conventional SL imaging at 3 T is highly challenging (49–52). RAFF2 requires less maximum RF power (≈30% lower SAR) when compared with a continuous wave pulse train of equal duration and equal RF pulse frequency (27). Thus, RAFF2 can be a SAR-efficient candidate for SL imaging, while potentially circumventing the SAR limitations of conventional SL imaging at 3 T. Moreover, compared to adiabatic ${\text{T}}_{1\rho }$, RAFF may more accurately reflect a single SL frequency component. This is because adiabatic ${\text{T}}_{1\rho }$ relaxation is influenced by the orientation of the magnetization, the effective field, and its strength, leading to variability during adiabatic ${\text{T}}_{1\rho }$ preparation pulses. In contrast, during RAFF preparations, the effective field strength and the fictitious field components responsible for the sweep remain constant and uniform throughout the preparation. Like ${\text{T}}_{1\rho }$, the RAFF method can selectively probe slow molecular motions of tissue water or proton chemical exchange. Despite both adiabatic ${\text{T}}_{1\rho }$ and ${\text{T}}_{\mathrm{RAFF}2}$ are based on relaxation during RF irradiation and operate within the same range of ${\text{B}}_{1}^{+}$, RAFF is conceptually distinct from continuous wave ${\text{T}}_{1\rho }$, adiabatic ${\text{T}}_{1\rho }$, and adiabatic ${\text{T}}_{2\rho }$. Unlike ${\text{T}}_{1\rho }$ and ${\text{T}}_{2\rho }$, where relaxation is governed exclusively by longitudinal or transverse relaxation, respectively, RAFF2 incorporates contributions from both ${\text{T}}_{1\rho }$ and ${\text{T}}_{2\rho }$ relaxations. This can result in lower relaxation rate constants with RAFF as compared to conventional off-resonance SL ${\text{T}}_{1\rho }$, which is especially beneficial at high magnetic fields where relaxation pathways such as anisochronous exchange are significantly accelerated (53). Nevertheless, adiabatic ${\text{T}}_{1\rho }$ and ${\text{T}}_{\mathrm{RAFF}2}$ may yield similar values and exhibit a correlation in certain applications.

The proposed sequence enabled successful acquisition of ${\text{T}}_{\mathrm{RAFF}2}$ mapping within a single BH manageable for patients. Sufficiently long RAFF2 preparations were achieved, rendering it suitable for use with clinical MRI systems. In this study, the optimal duration of the RAFF2 preparation block was determined by considering the inherent constraints of the scanner hardware and adhering to the SAR limit achievable within one cardiac cycle as well as RF duty cycle. The duration of the spoiler blips was aimed at mitigating image artifacts. It is important to note that adjustments to these durations may be required for different field strengths and scanner hardware configurations. While the obtained map quality was overall acceptable, residual off-resonance-induced artifacts were observed near the coronary sinus. Those artifacts are mostly restricted to the lateral wall, so evaluating septal ROIs, as commonly recommended for diffuse diseases, remains feasible (54). The off-resonance artifact observed in the vicinity of the coronary sinus is likely attributable to ${\text{B}}_{0}$ field inhomogeneities. Additionally, the presence of sub-optimal ${\text{B}}_{1}^{+}$ field intensifies the sensitivity to ${\text{B}}_{0}$ variations. The phantom results indicate moderate sensitivity of ${\text{T}}_{\mathrm{RAFF}2}$ times to ${\text{B}}_{0}$ and ${\text{B}}_{1}^{+}$ inhomogeneities. Considering the range of inhomogeneities typically observed in vivo [$\Delta \omega_{0}=\pm$200 Hz (55), $\eta_{1}$ = 0.5–0.7 (56–58)], this can account for significant deviations in ${\text{T}}_{\mathrm{RAFF}2}$ times. This study did not include ${\text{B}}_{0}$ and ${\text{B}}_{1}^{+}$ mapping in vivo. However, considering the residual sensitivity of ${\text{T}}_{\mathrm{RAFF}2}$ to field inhomogeneities, ${\text{B}}_{0}$ and ${\text{B}}_{1}^{+}$ mapping is crucial for future studies to allow for careful interpretation of ${\text{T}}_{\mathrm{RAFF}2}$ maps in vivo. To enhance robustness against ${\text{B}}_{0}$-related artifacts, RAFF2 pulses can be replaced with RAFFn pulses, where n>2 (28). As n increases in RAFFn, tolerance for both ${\text{B}}_{0}$ and ${\text{B}}_{1}^{+}$ inhomogeneities improves. Higher values of n result in significantly increased pulse bandwidth due to lower flip angles (59). Alternatively, a generalized inhomogeneity-resilient RAFF (girRAFF) pulse can be used to provide greater robustness in the presence of ${\text{B}}_{0}$ and ${\text{B}}_{1}^{+}$ field inhomogeneities (48). Additionally, in practical use, advanced high-order volumetric shimming can be applied to minimize off-resonance artifacts in the area of interest around the heart. Parallel RF transmission (pTx) techniques can also be employed to further reduce ${\text{B}}_{1}^{+}$-related artifacts, by means of static or dynamic pTx (60).

In patients, ${\text{T}}_{\mathrm{RAFF}2}$ time alterations were co-localized with hyperenhancement in LGE images, indicating the potential for MI detection. Patient 1 presented a left anterior descending coronary artery (LAD) territory infarct with a left ventricular apical thrombus and Patient 2 presented mid-RCA infarct. Infarcted areas showed higher ${\text{T}}_{\mathrm{RAFF}2}$ values than remote areas, which is in agreement with previous studies using ${\text{T}}_{\mathrm{RAFF}2}$ on 1.5 T (31) and 9.4 T (33). The increased tissue water content after a coronary artery occlusion, caused by extracellular space expansion or changes in proton chemical exchange, affects water-macromolecular interactions and may explain the observed increase in ${\text{T}}_{\mathrm{RAFF}2}$ in the infarcted area. The RAFF2 pulses operate in a sub-adiabatic condition with constant effective field strength and an identical, constant (stationary) fictitious field strength leading to uniform sweeps. This makes RAFF2 sensitive to intermediate and slow molecular motion, resulting in varying clinical sensitivity and higher scar-to-myocardium contrast than the laboratory frame relaxation time ${\text{T}}_{1}$, which is sensitive to molecular motion occurring at frequencies near the Larmor frequency. Nevertheless, to validate ${\text{T}}_{\mathrm{RAFF}2}$ mapping as a non-contrast scar evaluation tool for ischemic and non-ischemic heart disease with clinically acceptable SAR, further research is warranted. This proof-of-principle study included only two LGE-positive patients. The image acquisition was performed in different imaging views for the two patients, which is likely contributing to the differences observed in remote and infarct areas between the patients. Additionally, a discrepancy was observed between the average values of healthy subjects and the remote area of Patient 2. Therefore, larger cohorts of healthy controls and targeted patients are necessary to determine clinical sensitivity and establish cut-off values for distinguishing between remote and infarcted myocardium.

This proof-of-principle study has several limitations. We note that the phantom does not accurately represent tissue properties, as it lacks features like magnetization transfer and chemical exchange. Consequently, there is a conspicuous lack of realistic low frequency interactions, leading to the observed high correlation between RAFF2 and ${\text{T}}_{2}$, which does not necessarily hold in vivo. Moreover, histological validation after biopsy should also be included in future research. Furthermore, a direct comparison between ${\text{T}}_{\mathrm{RAFF}2}$, girRAFF, and other rotating frame relaxation methods, such as ${\text{T}}_{1\rho }$, was not conducted. High-rank RAFFn (n > 2) (28) may offer greater sensitivity to slow and ultra-slow molecular motions and reduce SAR, which could be beneficial for certain subjects or varying magnetic field strengths. Furthermore, advanced pTx systems can be used to achieve improved ${\text{B}}_{1}^{+}$ homogeneity, with the potential to substantially improve ${\text{T}}_{\mathrm{RAFF}2}$ map quality in vivo. Finally, inter-scan variability assessment over several days must be performed in future research.

In conclusion, myocardial ${\text{T}}_{\mathrm{RAFF}2}$ mapping was achieved with visually acceptable quality maps, largely homogeneous signal, and low variability. Myocardial infarction depiction was in agreement with LGE demonstrating the potential of non-contrast ${\text{T}}_{\mathrm{RAFF}2}$ mapping with clinically tolerable SAR. However, moderate off-resonance artifacts were present in some cases. Development of more inhomogeneity-resilient RAFF pulses as well as the evaluation of ${\text{T}}_{\mathrm{RAFF}2}$ in a larger patient cohort is warranted.

## Data Availability

The raw data supporting the conclusions of this article will be made available by the authors, without undue reservation.
