# Supplementary material for: Feasibility of relaxation along a fictitious field in the 2nd rotating frame (TRAFF2) mapping in the human myocardium at 3 T
Source: Front Cardiovasc Med. 2024 Dec 4;11:1373240. doi: 10.3389/fcvm.2024.1373240 (PMC11652659; doi:10.3389/fcvm.2024.1373240)
Supplement: Supplementary file 1 [file Image1.pdf]

Supplementary Material

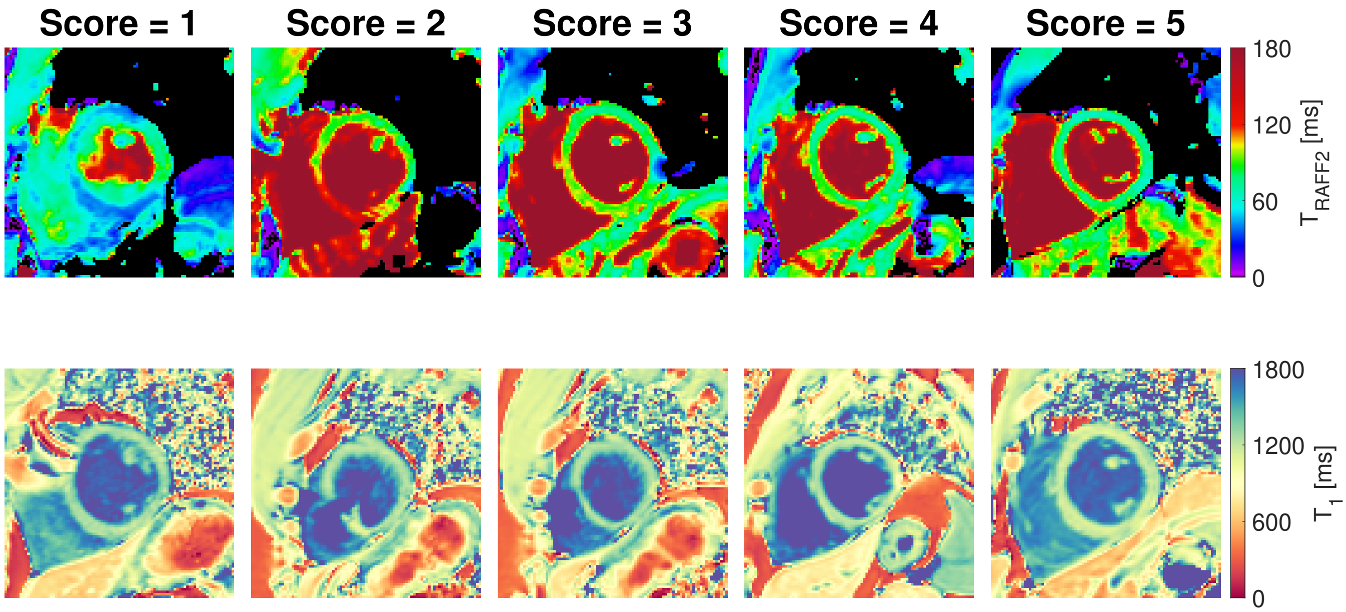

Figure S1. Representative T_{RAFF2} (top row) and T_1 (bottom row) maps for each image quality score.
